# Machine learning prediction of ACL loading during the wide lunge: a multifactorial coupling analysis based on kinematic and electromyographic signals

**DOI:** 10.3389/fbioe.2026.1880448

**Published:** 2026-07-29

**Authors:** Minting Fang, Yuhang Zhu, Guanzhong Wang, Qingqing Ma, Yanyu Yin, Yanlong Zhang, Si Chen

**Affiliations:** 1 College of Sports and Health Sciences, Mudanjiang Normal University, Mudanjiang, China; 2 Graduate School, Shenyang Sport University, Shenyang, China; 3 College of Physical Education, Shenzhen University, Shenzhen, China

**Keywords:** anterior cruciate ligament (ACL), badminton, explainable machine learning, wide lunge, XGBoost-SHAP

## Abstract

**Introduction:**

In badminton, the wide lunge is a common movement that is highly associated with anterior cruciate ligament (ACL) injury. Existing assessment methods mainly rely on subjective observation, three-dimensional motion capture systems, and surface electromyography signal acquisition, which makes it difficult to rapidly reveal the mechanisms of multi-joint coupling. This study developed and compared six machine learning (ML) algorithms extreme gradient boosting(XGBoost); gradient boosting decision tree (GBDT); random forest (RF); k-nearest neighbours (KNN); kernel ridge regression (KRR); support vector regression (SVR) to predict model-estimated ACL loading during the badminton wide lunge, expressed in multiples of body weight (BW). SHAP was used to rank feature importance and quantify feature contributions.

**Methods:**

A total of 237 amateur players were recruited, with kinematic and surface EMG data synchronously collected using a Qualisys motion capture system, AMTI force platforms, and a Delsys surface EMG system during the wide lunge. The sample-size sensitivity and overall predictive performance of the six machine-learning algorithms were evaluated.

**Results:**

XGBoost demonstrated the greatest robustness to sample size variation and achieved the best predictive performance (*R* ^*2*^ = 0.94, RMSE = 0.07, MAE = 0.05). Among the nine input features included in the XGBoost model, the SHAP global importance ranking was as follows: knee flexion angle (KFA), hamstrings-to-quadriceps ratio (H/Q), foot progression angle (FPA), internal tibial rotation angle (ITR), hip adduction angle (HAA), ankle dorsiflexion angle (ADF), trunk forward lean angle (TFA), knee valgus angle (KVA), and hip flexion angle (HFA) as the most influential features, with KFA (21.6%), H/Q (16.4%), and FPA (14.4%) contributing most.

**Conclusion:**

XGBoost effectively captured the complex non-linear relationships among biomechanical variables. Combined with SHAP-based explainability analysis, insufficient knee flexion, quadriceps-dominant neuromuscular control, and abnormal foot alignment were identified as a combined model-estimated high ACL loading pattern, suggesting that disrupted coupling between multi-joint coordination and muscle activation may be a key mechanism underlying increased injury risk. These findings may provide a basis for individualised training and injury prevention.

## 1 Introduction

The wide lunge is a common movement pattern in badminton and is typically executed under high-impact conditions. Similar to a rapid deceleration-and-landing task, it requires players to perform a continuous sequence of rapid push-off and braking upon landing on court (Kimura et al., 2010), and is therefore critical to match performance. It has been reported that the wide lunge accounts for more than 15% of all movements performed during a badminton match (Kuntze et al., 2010). However, this movement is also highly associated with anterior cruciate ligament (ACL) injury, particularly during the initial deceleration phase immediately after foot contact (Kaldau et al., 2024). Injury involves a multi-planar coupled biomechanical mechanism chain, including the coronal and transverse planes (KVA, ITR, and HAA), the sagittal plane (KFA, HFA, and TFA), distal ankle-foot mechanics (FPA and ADF), and neuromuscular control (H/Q). Previous studies have shown that knee valgus angle (KVA), internal tibial rotation angle (ITR), and hip adduction angle (HAA) determine the position of the femur relative to the tibia during the initial deceleration phase of landing and are directly associated with ACL injury mechanisms (Powers, 2010; Shin et al., 2011). In addition, a smaller knee flexion angle (KFA), greater knee valgus angle (KVA), and limited hip flexion angle (HFA) during landing have been identified as important indicators of increased ACL loading (Yeap et al., 2025). Meanwhile, trunk forward lean angle (TFA) and hip flexion angle (HFA) influence the position of the body centre of mass relative to the knee joint, thereby affecting lower-extremity load transmission patterns (Braun et al., 2025). Ankle dorsiflexion angle (ADF) and foot progression angle (FPA) reflect the distal ankle-foot support posture and the direction of foot alignment, which may influence the force transmission pathway of the knee joint (Lima et al., 2018). Collectively, these findings suggest that the dynamic coupling among lower-extremity kinematic variables determines the distribution of joint loading, reflecting the combined effects of multiple parameters. When any one of these parameters becomes abnormal, the line of force may be altered, thereby increasing the risk of ACL injury (Dadfar et al., 2021). In addition to kinematics, neuromuscular control, as reflected by the hamstrings-to-quadriceps co-activation ratio (H/Q), exhibits complex coupling with kinematic parameters such as knee flexion angle (KFA), hip adduction angle (HAA), and foot progression angle (FPA). When the H/Q is excessively high or low, the muscular regulatory mechanism may be weakened. This adverse temporal coupling between kinematic and electromyographic signals has been identified as being associated with ACL loading and injury risk (Hannah et al., 2014). Compared with abnormalities in a single variable, such complex coupling interactions may better reveal the underlying nature of joint loading risk. Thus, adverse multifactorial coupling between kinematic and electromyographic signals during wide lunge landing may represent a potential biomechanical marker of joint loading and injury risk. Therefore, predicting high-load movement patterns based on biomechanical parameters characterised by multifactorial coupling across the three anatomical planes (sagittal, coronal, and transverse planes), distal ankle-foot mechanics (FPA and ADF), and neuromuscular control (H/Q) may further clarify the relationship between these parameters and joint loading, thereby helping to inform the prevention of potential ACL injury during wide lunge landing.

In sports involving abrupt deceleration and side-cut landing tasks, the most commonly used approaches for predicting ACL injury risk are kinematic analyses based on two-dimensional (2D) and three-dimensional (3D) motion capture systems (Xie et al., 2025). These methods are mainly characterised by the indirect assessment of injury risk through quantification of joint angles at the instant of initial contact, which makes them suitable for clinical application and large-scale screening. At present, laboratory-acquired parameters, including kinematic, kinetic, and surface electromyographic signals, are commonly used to establish linear relationships using models such as ordinary least squares (OLS), Elastic Net, and logistic regression. These linear models typically predict risk based on a single risk indicator under a linearity assumption (Roustaei, 2024). In model learning, predictor effects are assumed to be purely additive and slopes remain constant, making these models unable to capture parameter interactions, non-linear characteristics, or threshold turning points (Zhou et al., 2023). Beyond laboratory-based measurements, the Landing Error Scoring System (LESS) is a clinical screening tool used to assess jump-landing movement quality and identify ACL injury risk. It qualitatively evaluates landing posture by observing participants performing a standardised jump–landing–rebound task and scoring variables such as knee valgus, hip flexion, trunk flexion, and foot landing position (Padua et al., 2015). This tool is easy to administer and requires only two-dimensional video capture, and it has shown good reliability and validity in athlete injury-prevention screening. However, the scoring process still depends on the evaluator’s subjective judgement of joint angles and trunk posture from 2D video images. It also cannot reflect the complex temporal characteristics of real sporting movements, such as abrupt stopping, change of direction, side-cutting, and single-leg landing, and may therefore underestimate the interaction mechanisms underlying injury risk in actual training and competition (Smith et al., 2012). Thus, this approach is highly subjective, does not allow quantitative analysis of the assessment results, and is difficult to use for capturing kinematic changes and muscle activation at foot contact (Hanzlíková and Hébert-Losier, 2020). This highlights the need to develop an integrated feature model capable of capturing multi-parameter interactions to improve the accuracy and scientific validity of individualised ACL injury risk prediction.

Machine learning (ML) models have increasingly been developed to reveal complex non-linear relationships among input features. By automatically learning patterns from data, these algorithms can construct predictive models for new data and have been widely applied across multiple fields (Leckey et al., 2025). Commonly used ML algorithms include extreme gradient boosting (XGBoost), gradient boosting decision tree (GBDT), random forest (RF), k-nearest neighbours (KNN), kernel ridge regression (KRR), and support vector regression (SVR). When combined with SHapley Additive exPlanations (SHAP), these models can be used to interpret prediction outcomes by identifying globally important features and providing local explanations, thereby capturing complex non-linear biomechanical characteristics associated with injury (Aghababa and Andrysek, 2024). Recent applications of ML in sport have included performance analysis, injury prediction, tactical decision support, and training optimisation (Reis et al., 2024; Yuan et al., 2026). Related studies have shown that, by integrating multi-source data such as physiological signals, psychological signals, and training context, ML can generate predictive models that outperform traditional approaches (Jianjun et al., 2025), and can be applied to tennis point outcome prediction, athlete performance evaluation, and injury risk prediction in athletes (Bozděch and Zháněl, 2023; Li, 2025; Ren et al., 2025). However, these studies have mainly focused on sports performance or general injury risk prediction, while their explanation of injury-related biomechanical mechanisms remains limited. In recent years, AI-driven biomechanical modelling, through the integration of computer vision, wearable technology, and deep learning, has shown potential for injury prevention and sports performance improvement (Chen et al., 2026; Souaifi et al., 2025), providing a new technical pathway for movement pattern recognition and injury prediction. In addition, in tasks such as stair descent or landing, the dynamic changes in different muscle groups may be closely related to multi-muscle coordinated control strategies, suggesting that lower-limb stability does not depend only on a single kinematic variable but may result from the combined effects of kinematics and neuromuscular control (Wei et al., 2026; Jie et al., 2024). However, previous studies have mainly focused on daily tasks such as walking and stair descent, with insufficient attention paid to sport-specific movements characterized by multifactorial coupling, such as the badminton wide lunge. ML-based prediction of ACL loading during the wide lunge using kinematic and electromyographic signals remains rarely reported. Therefore, this study aimed to quantify biomechanical features using kinematic analysis, musculoskeletal modelling and simulation, and sEMG, and to develop and compare ML models based on different algorithms. The best-performing model was then selected to reveal the multifactorial coupling mechanisms between kinematic and electromyographic signals associated with ACL injury. Furthermore, a web-based application for ACL risk prediction based on multifactorial coupling was developed to provide visualised and individualised support for clinical prevention. On this basis, we hypothesised that XGBoost would outperform the other models in capturing complex multifactorial biomechanical interactions; that increased ACL loading during the wide lunge would arise from the coupling of multiple parameters, particularly knee flexion angle, muscle co-activation ratio, and foot position; and that ML-based prediction of ACL loading during the wide lunge would have practical value at the individual level.

## 2 Methods

### 2.1 Participants and testing procedures

**FIGURE 1:**
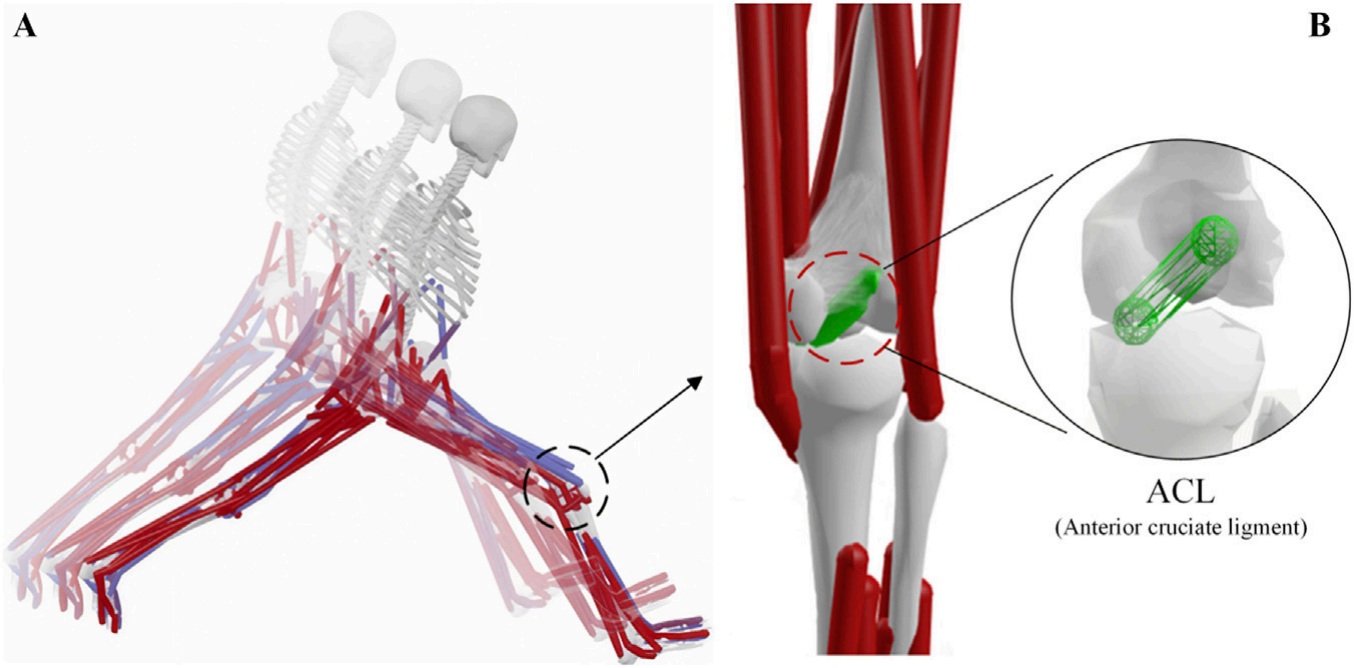
Import the 2392ACL model into OpenSim for musculoskeletal simulation modeling. **(A)** OpenSim musculoskeletal model during the wide lunge movement. **(B)** Enlarged knee joint view showing the modelled anterior cruciate ligament in the 2392ACL model.

A total of 237 amateur badminton players were recruited (male; age: 21.67 ± 1.37 years; height: 1.71 ± 0.06 m; body mass: 69.83 ± 3.71 kg), all of whom were right-hand dominant. Inclusion criteria were at least 3 years of badminton experience, participation in training or competition 2–3 times per week, no upper- or lower-extremity injury within the previous 6 months, and no high-intensity training or competition within 48 h before testing. The study was approved by the Ethics Committee of Mudanjiang Normal University (No. 2025008), and written informed consent was obtained from all participants. In Figure 1A, the experimental task was a badminton wide lunge with stroke execution. The force platform was positioned in the right-front direction of the participant at a distance of 1.5 times leg length, with the movement direction oriented 45° to the anterior–posterior axis of the platform. A shuttlecock was suspended 0.6 m above the ground on the right side of the platform to simulate a game-like wide lunge stroke.

In this study, the prediction model sample size framework proposed by Riley et al. (2020) was used to assess the adequacy of the available sample size for model development. The calculation was performed using the Stata package pmsampsize (version 1.3.2). The model included nine candidate predictors, with the anticipated shrinkage factor set at 0.90 and the multiplicative margin of error (MMOE) for intercept estimation set at 1.10. The minimum sample size required to control model overfitting was 37 participants, whereas the sample size required for precise estimation of the residual standard deviation and mean outcome was 243 participants. Each participant contributed only one valid wide-lunge trial. The 237 participants included in this study yielded an estimated MMOE of approximately 1.101 for the residual standard deviation, indicating that the sample size was slightly below the precision criterion but satisfied the requirement for controlling model overfitting.

### 2.2 Data collection and phase definition

Thirty-nine retroreflective markers were placed according to the Plug in Gait model. Kinematic data were captured using an eight-camera infrared motion capture system (Qualisys 600+) at 200 Hz and synchronized with force plate data (BP400600, AMTI) sampled at 1,000 Hz. sEMG was recorded using a 16-channel wireless system (Delsys) at 2,000 Hz from the right rectus femoris, vastus lateralis, vastus medialis, semitendinosus, biceps femoris, tibialis anterior, and lateral gastrocnemius. Based on evidence that ACL injury typically occurs within the first 50 ms after initial ground contact during landing tasks (Bates et al., 2020), analyses focused on the 0–50 ms interval following heel contact with the force plate. Initial contact was defined as the instant when the vertical ground reaction force first exceeded 10 N after right heel contact (Walsh et al., 2012). Participants were instructed to land within the central region of the force plate.

### 2.3 Musculoskeletal simulation and sEMG processing

Musculoskeletal simulations were performed using an upgraded OpenSim Gait2392_simbodyadd_ACL model, in which an ACL component was incorporated into the original Gait2392 lower-extremity model, as shown in Figure 1B. The model included the trunk and back joints (Delp et al., 1990) and comprised 12 rigid segments, 23 degrees of freedom, and 92 muscle actuators. The geometric and mechanical characteristics of the ACL were represented by 10 fibre bundles (Xu et al., 2015). Within the coordinate system framework of the generic Gait2392 model, the attachment point coordinates of the ACL fibre bundles were defined with reference to the cadaveric knee experimental data reported by Blankevoort and Huiskes (1991). The OpenSim Scale Tool was used to scale the bone dimensions, joint positions, and the femoral and tibial attachment point locations of the ACL according to each participant’s static marker data. The global coordinates of the attachment points were obtained by multiplying the local coordinates by the scaling matrix of the corresponding bone, thereby enabling participant-specific localization of the ACL attachment points.

The length of each ACL fibre bundle followed the geometric definition of the knee ligament model in OpenSim (Seth et al., 2018). The instantaneous length at each time point was calculated as the Euclidean distance between the femoral and tibial attachment points after scaling and transformation into the global coordinate system:

$$l_{i}\left(t\right)=\left\|T_{f}^{G}\right.\left(t\right)S_{f}r_{f,i}^{local}-T_{T}^{G}\left(t\right)S_{t}\left.r_{t,i}^{local}\right\|$$ (1)

where $r_{f,i}^{local}$ and $r_{t,i}^{local}$ denote the local coordinates of the femoral and tibial attachment points, respectively, of the (i)-th ACL fibre bundle in the unscaled model; $S_{f}$ and $S_{t}$ denote the scaling matrices of the femur and tibia, respectively; $T_{f}^{G}\left(t\right)$ and $T_{T}^{G}\left(t\right)$ denote the pose transformation matrices of the femur and tibia relative to the global coordinate system at time (t), respectively; and $\left\|\cdot \right\|$ denotes the Euclidean norm.

The slack length was defined as the initial length of each ACL fibre bundle in the reference posture of the participant-scaled model. Full knee extension was used as the reference posture, and the length of each ACL fibre bundle was recalculated based on the scaled femoral and tibial attachment point coordinates. This recalculated length was defined as the slack length of the corresponding fibre bundle (Shelburne et al., 2006).

The strain of each bundle was defined as follows:

$$\varepsilon =\frac{l-l_{slack}}{l_{slack}}$$ (2)

The mechanical behaviour of the ligament was described using the Blankevoort ligament model, in which each ligament bundle was defined as a non-linear elastic and determined by ligament strain, slack length, and stiffness parameters. Ligament force ( F ) was expressed as a piecewise non-linear function:

$$F=\left\{\begin{array}{c} \frac{1}{4\varepsilon_{l}}k\varepsilon^{2},0<\varepsilon <2\varepsilon_{l}\\ k\left(\varepsilon -\varepsilon_{l}\right),\varepsilon \geq 2\varepsilon_{l}\\ 0,\varepsilon \leq 0 \end{array}\right.$$ (3)

where F denotes the ligament bundle tension; ε denotes the ligament bundle strain; k denotes the ligament bundle stiffness parameter; and $\varepsilon_{l}$ denotes the strain parameter controlling the nonlinear toe region. The ligament force–strain relationship was based on the nonlinear elastic ligament model proposed by Blankevoort and Huiskes. The stiffness values and reference strain were adopted from the parameter settings of a three-dimensional knee musculoskeletal model to ensure that the passive ligament constraint properties of the model were consistent with those of the established three-dimensional knee model (Wang et al., 2015).

Muscle actuation was modelled using the classical Hill-type muscle model, with key parameters including maximum isometric force ( $F_{\max}$ ), optimal fibre length ( $l_{opt}$ ), tendon slack length ( $l_{slack}$ ), and the force–length and force–velocity relationships, which together determine muscle force-generating capacity (Cowburn et al., 2024). Simulations were performed in OpenSim 4.4. First, model scaling was conducted to match each participant’s anthropometrics using the automatic scaling tool (AST) (Di Pietro et al., 2024), a MATLAB script based on the OpenSim API that iteratively combines static marker scaling and inverse kinematics and applies RMSE-based corrections to markers that fail quality checks, thereby reducing scaling-related errors. Inverse kinematics (IK) was then performed to estimate joint angles, followed by the residual reduction algorithm (RRA) to improve dynamic consistency between model kinematics and ground reaction forces/moments. Finally, computed muscle control (CMC) was used to estimate muscle activations and ACL loading. To minimise inter-individual variability, ACL loading was normalised to body weight and expressed in body weights (BW).

sEMG data were exported from Qualisys as TSV files and converted to plain-text format. All processing was performed in MATLAB R2023a. Before the formal testing, maximal voluntary isometric contractions (MVCs) of each target muscle were collected for subsequent normalization of sEMG amplitude. Before data collection, participants were instructed to perform each task three times, with each contraction lasting 5 s and a 10 s rest interval between trials. The testing procedures were as follows. The MVC of the quadriceps was elicited through isometric knee extension. Participants were seated with the hip and knee flexed at approximately 90°, the distal shank fixed, and were instructed to perform a maximal knee extension effort (Ekstrom et al., 2012). The MVC of the biceps femoris was elicited through isometric knee flexion. Participants were also seated with the knee maintained at approximately 90° of flexion, the distal shank fixed, and were instructed to perform a maximal knee flexion effort (Kirk and Rice, 2017). The MVC of the gastrocnemius was elicited through isometric ankle plantarflexion. Participants stood with the ankle maintained in a neutral position and performed a maximal ankle plantarflexion contraction with the foot fixed or externally resisted (Schwartz et al., 2020). The MVC of the tibialis anterior was elicited through isometric ankle dorsiflexion. Participants were seated with the ankle maintained in a neutral position, external resistance was applied to the dorsum of the foot, and participants were instructed to perform a maximal ankle dorsiflexion effort (Ugbolue et al., 2021). Raw sEMG were filtered using a fourth-order 20–500 Hz Butterworth filter, and the RMS envelope was calculated using a 50 ms moving window. For the MVC test of each muscle, a 1 s time window was selected during the stable maximal contraction phase to calculate the mean RMS value, and the highest mean RMS value across the three MVC trials was used as the MVC reference value for that muscle. Subsequently, the sEMG amplitude of the corresponding muscle during the task was processed using the same method and divided by the MVC reference value of that muscle, and was expressed as %MVC (Schwartz et al., 2020). Muscle co-activation ratios were computed using RMS values: *RMS*_*Antagonist*_ */RMS*_*Agonist*_.

**FIGURE 2:**
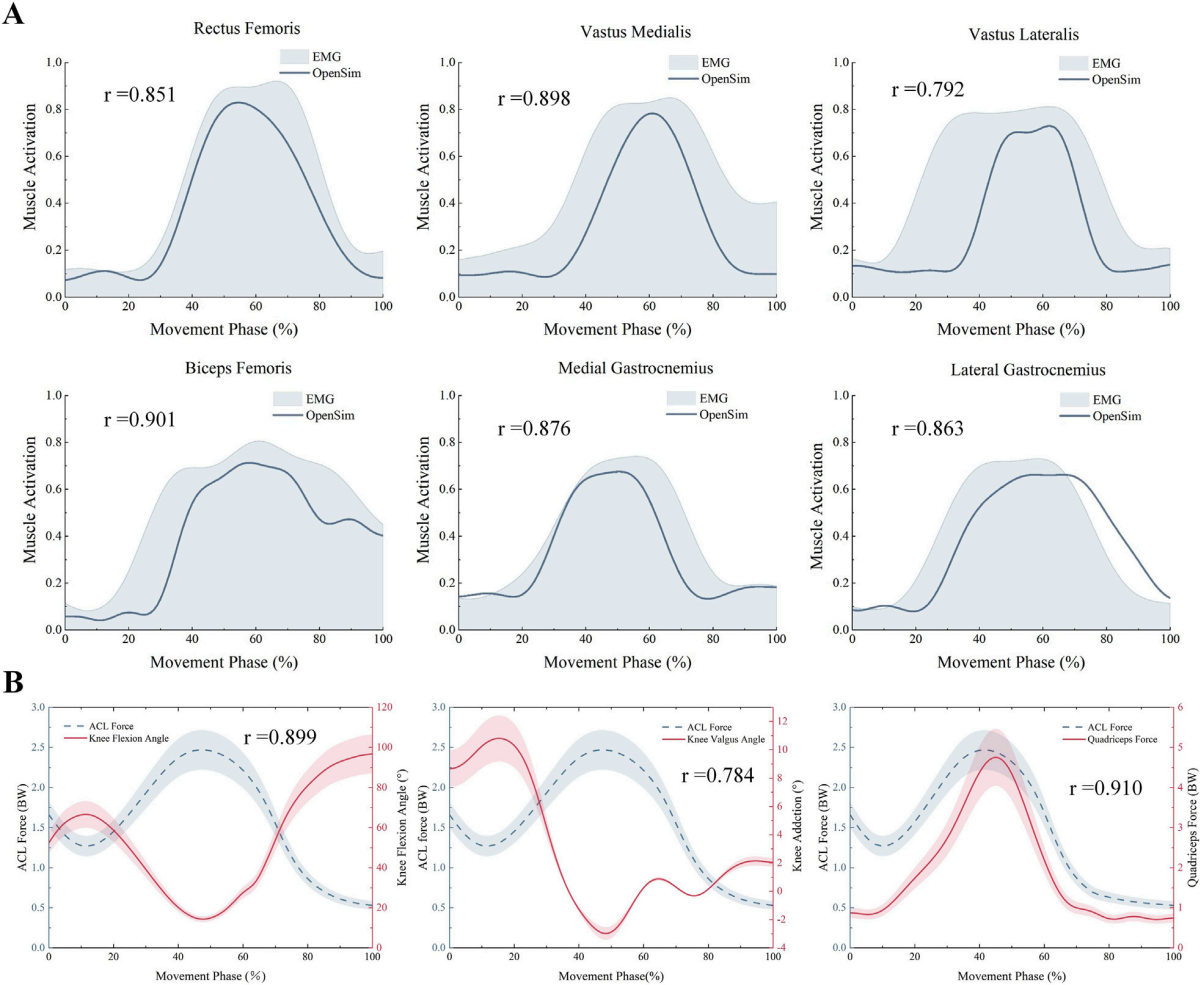
**(A)** Comparison of surface EMG signals and OpenSim-simulated muscle activation curves; **(B)** relationship between kinematic/neuromuscular variables and OpenSim-estimated ACL loading. Correlation coefficients (r) are shown for each subplot.

Model validation consisted of two components: muscle activation validation and ACL force trend validation, as shown in Figure 2. The surface electromyography signals of the main muscles during the badminton wide lunge were compared with the OpenSim-simulated muscle activation curves in terms of waveform trends to evaluate the reasonableness of the simulated muscle activation patterns. Meanwhile, ACL force was compared with the curves of KFA, KVA, and quadriceps muscle force in terms of trend consistency (Hannah et al., 2014; Englander et al., 2019; Ogasawara et al., 2024), in order to assess whether the changes in ACL force were consistent with the physiological principles of knee joint load transmission and ACL loading. To further evaluate the reasonableness of the ACL force calculated by the musculoskeletal model, the peak ACL force obtained from the simulation was compared with the ACL loading ranges reported in previous experimental studies of high-risk movements, such as landing and cutting. Previous studies have reported that ACL loading during landing and cutting movements is approximately 2.3 BW (Moon et al., 2023; Nasseri et al., 2021). Therefore, this range was used as an external reference in the present study to determine whether the simulated ACL force was within a physiologically reasonable range.

### 2.4 Statistical analysis

Pearson correlation analysis was used for preliminary feature selection. The dataset was divided into training and test sets using a fixed random seed (random_state = 42). Feature selection was performed only on the training set, whereas the test set was used only for final model performance evaluation. An absolute correlation coefficient of (|r| < 0.30) is generally considered to indicate a weak or negligible correlation, and variables with (|r| < 0.30) were therefore excluded (Mukaka, 2012).

Descriptive statistics for the nine selected input features and the ACL loading output variable were computed using SPSS (version 28.0), including minimum, maximum, mean, and standard deviation. The distributional characteristics of each variable were assessed using the Shapiro–Wilk test, with the significance level set at α = 0.05. As this part of the study was primarily intended to describe sample characteristics, the descriptive results are presented as mean ± standard deviation, together with the corresponding minimum and maximum values.

### 2.5 Machine learning modelling and evaluation

To develop the ACL risk prediction model, six ML algorithms were compared: extreme gradient boosting (XGBoost), gradient boosting decision tree (GBDT), random forest (RF), k-nearest neighbours (KNN), kernel ridge regression (KRR), and support vector regression (SVR).

GBDT is an ensemble method that combines gradient boosting with regression trees by sequentially training new weak learners to fit residuals, thereby progressively improving model performance (Zhang et al., 2019). XGBoost extends GBDT by incorporating L1 and L2 regularization and iteratively growing CART subtrees along the negative-gradient direction to minimize the objective function. It applies explicit regularization to both the tree structure and leaf weights to control overfitting, and uses a second order Taylor expansion of the loss to include second order gradients in the optimization, improving accuracy and stability (Chen et al., 2016).

RF is an ensemble learning method composed of decision trees that adopts a typical bagging strategy. For regression, it repeatedly draws random training subsets from the original dataset to train multiple trees, and averages the outputs of all trees at inference to obtain the final prediction (Hu and Szymczak, 2023). However, because each tree is trained independently, RF lacks the stage-wise residual fitting capability used by XGBoost and GBDT.

$$\text{XGBoost}:{\;\tilde{L}}^{\left(t\right)}=\sum_{i=1}^{n}\left[g_{i}f_{t}\left(x_{i}\right)+\frac{1}{2}h_{i}f_{t}{\left(x_{i}\right)}^{2}\right]+\Omega \left(f_{t}\right)+const$$ (4)

$$\text{RF}:\hat{y}=\frac{1}{T}\sum_{t=1}^{T}f_{t}\left(x\right)$$ (5)

KNN is a classical non-parametric algorithm used for both classification and regression. In KNN regression, distances between samples are computed in the feature space to select the k nearest neighbors. For each sample, the prediction is obtained as a weighted average of the response values of these k nearest samples in the dataset, and this average is taken as the predicted output (Srisuradetchai and Suksrikran, 2024).

$$\hat{y}\left(x\right)=\frac{\sum_{i\in N_{k}\left(x\right)}w_{i}\left(x\right)y_{i}\;}{\sum_{i\in N_{k}\left(x\right)}w_{i}\left(x\right)}$$ (6)

KRR is an extension of classical linear ridge regression that incorporates a kernel function. The kernel maps the original input features, which are limited to linear regression in the input space, into a higher dimensional feature space. Directly constructing a regression function in this high-dimensional space would be computationally expensive. The kernel trick allows the model to be expressed in terms of kernel evaluations between samples, avoiding explicit computations in the high-dimensional feature space while enabling nonlinear regression (Chu et al., 2011). This approach can yield regression models with good predictive performance.

$$\hat{y}\left(x\right)=\sum_{i=1}^{n}\alpha_{i}k\left(x_{i},x\right)=k{\left(x\right)}^{⊺}{\left(Κ+⋋Ι\right)}^{-1}y$$ (7)

SVR is the regression extension of the Support Vector Machine (SVM). It adopts an ε-insensitive loss function, penalizing samples whose residuals fall outside an ε-tube around the regression function, thereby controlling the magnitude of prediction errors. By using kernel functions, SVR implicitly maps inputs into a higher-dimensional feature space and fits a linear model in that space (Shoko and Sigauke, 2023). As a result, SVR often exhibits stronger generalization and greater stability than conventional linear models.

$$f\left(x\right)=\sum_{i=1}^{n}\left(\alpha_{i}-\alpha_{i}^{\backslash *}\right)k\left(x_{i},x\right)+b$$ (8)

Using Python 3.13, the 237 samples were split into a training set (80%, N = 189) and an independent test set (20%, N = 48). Each participant contributed only one eligible badminton wide-lunge trial, and the samples in the training and test sets were derived from different participants. The training set was used for model development and cross-validated hyperparameter tuning, whereas the test set was used as an independent dataset to evaluate model performance under each data split. In this study, 50 repeated random data splits were used to evaluate the predictive stability of the six machine learning models. In each split, 80% of the samples were used as the training set, and the remaining 20% were used as an independent test set. Hyperparameter optimisation was conducted using a Bayesian optimisation approach based on the Tree-structured Parzen Estimator (TPE), implemented via the Optuna framework. This models the conditional distributions of hyperparameters and partitions previously evaluated configurations into good and poor groups, thereby favouring better-performing hyperparameter combinations (Zhivkov and Kandilarov, 2026). For each model, 50 hyperparameter optimisation trials were performed (n_trials = 50). The mean *R* ^*2*^ from five-fold cross-validation within the training set was used as the optimisation objective to identify the optimal hyperparameter combination. After the optimal hyperparameters were obtained for each random split, the model with the optimal parameters was retrained on the complete training set and then evaluated on the corresponding independent test set using R^2^, RMSE, and MAE. Test-set performance was expressed as the mean ± standard deviation across the 50 repeated random splits. Model performance was considered good when *R* ^*2*^ was ≥0.80 and both RMSE and MAE approached zero (Chicco et al., 2021). The best-performing model was ultimately selected for ACL load prediction. To evaluate the internal stability of the models, 250 cross-validation performance values were obtained for each model (50 random splits × 5 folds). Cluster bootstrap resampling was performed 10,000 times to calculate the 95% confidence intervals for cross-validation performance. In addition, a representative model trained using a fixed random seed (random_state = 42) was used for learning-curve diagnostics and SHAP interpretation analysis to ensure the reproducibility of the visualisation results (Sivakumar et al., 2024).

### 2.6 SHAP interpretation framework and feature collinearity diagnostics

Before SHAP interpretation, the variance inflation factor (VIF) was used to assess multicollinearity among the input biomechanical variables. VIF analysis was performed using the variance inflation factor function in statsmodels. Each input feature was sequentially treated as the dependent variable, while the remaining input features were used as explanatory variables in a linear regression model, in order to evaluate the overall degree of linear dependence between that feature and the other features. The VIF was calculated as follows:

$$VIF_{i}=\frac{1}{1-R_{i}^{2}}$$ (9)

where $R_{i}^{2}$ denotes the coefficient of determination obtained when the (i)-th input feature is explained by the remaining input features. Previous studies have shown that VIF <5 is considered to be within an acceptable range, whereas VIF >10 indicates serious multicollinearity (Kim, 2019).

SHAP was used to interpret the regression outputs of the XGBoost model. SHAP computes Shapley values from cooperative game theory to explain individual predictions by quantifying each feature’s contribution, thereby translating black-box outputs into interpretable feature attributions. In this framework, the Shapley value provides an additive feature attribution for a given instance (Lundberg et al., 2020). SHAP is defined as:

$$\emptyset_{j}\left(x\right)=\sum_{S\subseteq F\backslash \left\{j\right\}}\frac{\left|S\right|!\left(M-\left|S\right.\left|-1\right.\right)!}{M!}\left[f_{S\cup \left\{j\right\}}\left(x_{S\cup \left\{j\right\}}\right)-f_{S}\left(x_{S}\right)\right]$$ (10)

Let F denote the full feature set and M = |F| the total number of features. Let $f_{S}\left(x_{S}\right)$ represent the model output for sample x when only the features in subset S⊆F are used. The Shapley value $\emptyset_{j}\left(x\right)$ quantifies the contribution of the j th biomechanical feature to the predicted ACL loading, where positive and negative values indicate increased and decreased predictions, respectively. By aggregating | $\emptyset_{j}\left(x\right)$ | and examining interaction effects $\emptyset_{ij}\left(x\right)$ , global analyses identified key factors and their interactions, helping to interpret model performance and the potential biomechanical mechanisms underlying each feature’s effect.

**FIGURE 3:**
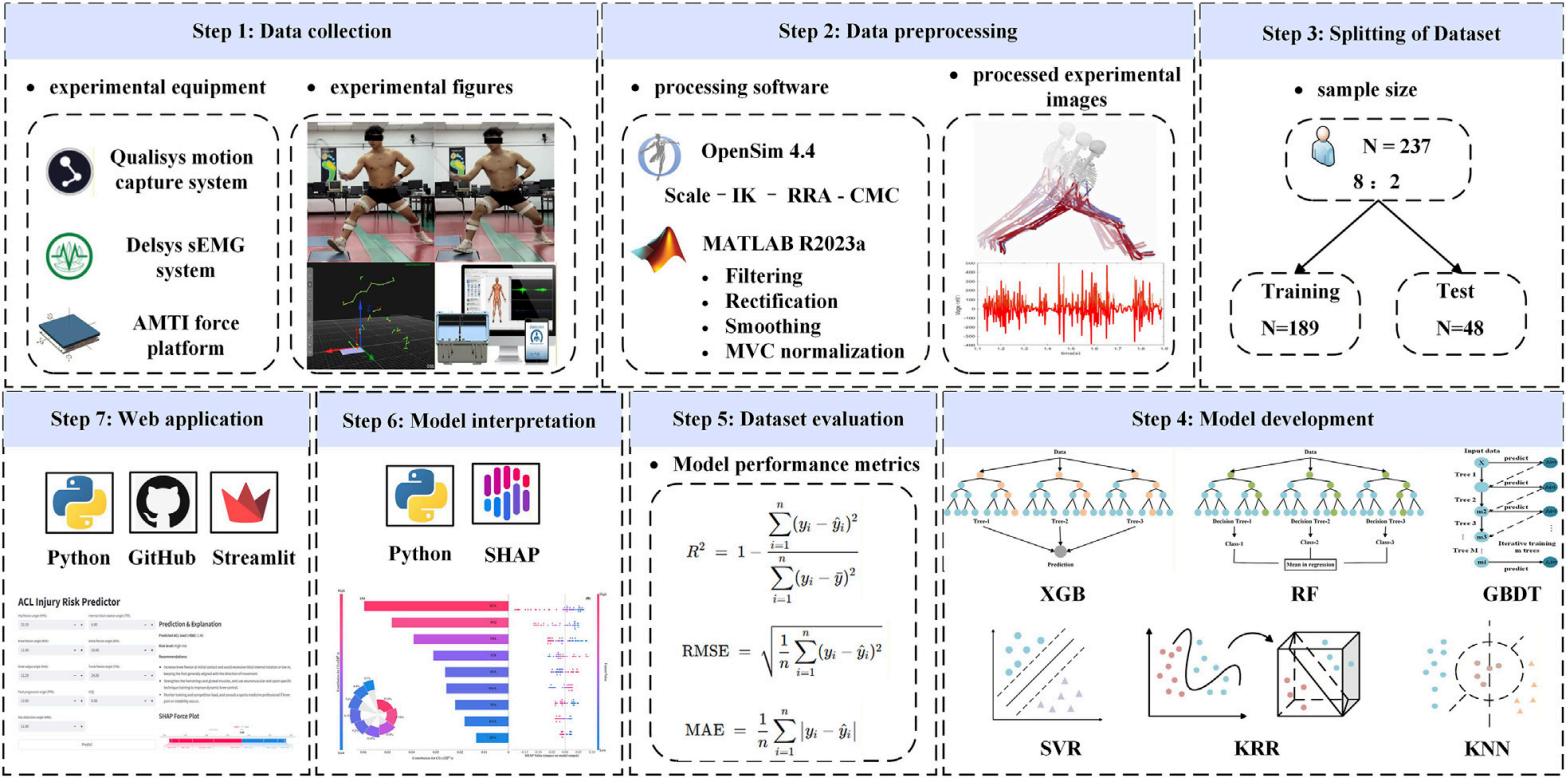
Overall experimental workflow of the study.

Figure 3 outlines the overall methodological workflow of this study. The pipeline comprises seven sequential steps: data acquisition, data preprocessing, dataset splitting, model development, model performance evaluation, model interpretation, and web application deployment. This workflow follows the order of the Methods section and provides a clear overview of the study’s technical framework.

### 2.7 Web-based application deployment

After the final XGBoost regression model was established, the web-rendering scripts, the trained model file, and all required package dependencies were organised and uploaded to a GitHub repository. Streamlit was then used to connect to the GitHub repository and integrate the model, frontend and backend code, and runtime environment into a web-based application, thereby completing deployment of the ACL load prediction platform.

## 3 Results

### 3.1 Feature selection and descriptive statistics of the dataset

As shown in Table 1, most biomechanical parameters were correlated with ACL force to varying degrees. Among them, HRA (r = −0.208) and TA/GL (r = −0.201) showed weak correlations with ACL force and did not meet the predefined selection criterion. Combined with the VIF analysis, nine features were finally selected for subsequent machine learning modelling: HFA, HAA, KFA, ITR, KVA, ADF, FPA, TFA, and H/Q.

**TABLE 1:** Pearson correlation analysis for candidate feature selection.

Parameters	Correlation coefficient
HFA	−0.673
HRA	−0.208
HAA	0.319
KFA	−0.831
ITR	0.823
KVA	−0.600
ADF	0.388
FPA	−0.753
TFA	−0.456
H/Q	−0.844
TA/GL	−0.201

A dataset was constructed by combining OpenSim-simulated ACL loading and kinematic variables with surface EMG measures, and the complete dataset is provided in the Supplementary Material. ACL loading served as the regression target (Y), and the remaining variables were used as input features (X). Samples were randomly split 8:2 into a training set (n = 189) and a test set (n = 48) for XGBoost model training and evaluation. After model-independent feature selection, the features used for model development were hip flexion angle (HFA), hip adduction angle (HAA), knee flexion angle (KFA), tibial internal rotation angle (ITR), knee valgus angle (KVA), ankle dorsiflexion angle (ADF), foot progression angle (FPA), trunk forward lean angle (TFA), and hamstrings-to-quadriceps ratio (H/Q). The standardization procedure is described in the Methods section. The minimum, mean, maximum, and standard deviation of the input features and target variable are summarized in Table 2.

**TABLE 2:** Input features and output variable in the training and test sets.

Model	Training				Testing			
	Minimum	Average	Maximum	Standard deviation	Minimum	Average	Maximum	Standard deviation
HFA	3.98	30.05	52.99	11.67	5.71	30.36	51.50	10.91
HAA	−20.89	13.17	27.95	8.39	−5.82	11.95	25.47	8.97
KFA	4.70	24.21	46.77	8.62	8.10	25.36	57.69	9.14
ITR	−19.84	2.06	17.41	7.33	−21.17	1.94	12.70	7.34
KVA	−16.26	2.77	18.38	8.51	−11.29	5.05	18.02	7.47
ADF	−22.85	8.06	36.25	15.27	−17.35	3.84	32.57	14.81
FPA	−4.14	9.77	22.26	5.53	2.26	10.35	22.85	5.05
TFA	9.46	26.60	45.09	8.11	14.02	27.06	43.02	8.95
H/Q	0.35	0.64	0.98	0.11	0.46	0.66	1.03	0.12
ACL	1.79	2.41	2.82	0.26	1.71	2.38	2.85	0.28

Positive values indicate flexion for HFA, KFA, and ADF; adduction for HAA; internal rotation for ITR; toe-out for FPA; and forward trunk lean for TFA., Negative KVA, values indicate knee valgus.

### 3.2 Model performance comparison

#### 3.2.1 Hyperparameter optimisation

Table 3 presents the results of hyperparameter optimisation for the ML algorithms using Bayesian optimisation. For GBDT (*R* ^*2*^ = 0.88), the optimal hyperparameter combination was *n_estimators* (ne) = 194, *learning_rate* (Lr) = 0.05, and *max_depth* (MD) = 3. For XGBoost (*R* ^*2*^ = 0.89), the optimal configuration was ne = 175, Lr = 0.06, and MD = 4; in addition, the inclusion of the regularisation parameters *reg_alpha* = 0.03 and *reg_lambda* = 1.20 further improved model generalizability. RF (*R* ^*2*^ = 0.88) achieved optimal performance with ne = 189, MD = 6, *min_samples_leaf* = 2, and *max_features* = sqrt. For KRR (*R* ^*2*^ = 0.86), the radial basis function (*rbf*) kernel was selected as optimal, with *alpha* = 0.05 and *gamma* = 0.001. SVR (*R* ^*2*^ = 0.83) performed best with *C* = 5.01 and *gamma* = 0.007, and the *rbf* Kernel was used. KNN (*R* ^*2*^ = 0.87) achieved optimal performance with *n_neighbors* = 4 using the *Minkowski* distance metric.

**TABLE 3:** Bayesian optimization results of hyperparameters.

Model	Hyperparameter	Search range	Best trial	Optimal value	CV R^2^
GBDT	n_estimators	[50, 200]	44	194	0.88
	learning_rate	[0.01, 0.1]		0.05	
	max_depth	[2, 6]		3	
XGBoost	n_estimators	[50, 200]	26	175	0.89
	learning_rate	[0.01, 0.1]		0.06	
	max_depth	[2, 6]		4	
	reg_alpha	[0.001, 0.5]		0.03	
	reg_lambda	[0.5, 2.0]		1.20	
RF	n_estimators	[50, 200]	42	189	0.88
	max_depth	[2, 6]		6	
	min_samples_leaf	[2, 8]		2	
	max_features	sqrt		sqrt	
		log2			
		1.0			
KRR	kernel	rbf	42	rbf	0.86
		linear			
	alpha	[0.05, 5.0]		0.05	
	gamma	[0.001, 0.1]		0.001	
SVR	C	[5.0, 500.0]	38	5.01	0.83
	gamma	[0.0001, 0.1]		0.007	
	kernel	poly		rbf	
		rbf			
		sigmoid			
KNN	n_neighbors	[1, 15]	16	4	0.87
	Distance metric	Minkowski		Minkowski	
		Euclidean			
		Manhattan			

#### 3.2.2 Performance across sample sizes

**FIGURE 4:**
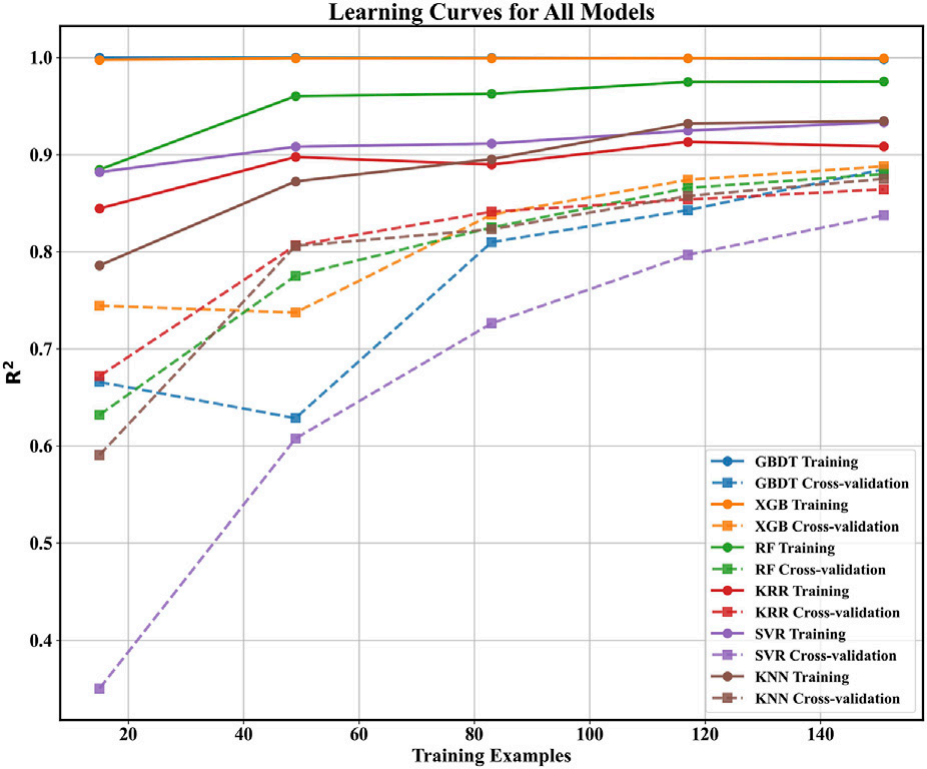
Cross-validated learning curves depicting model performance as a function of training sample size. The horizontal and vertical axes represent the number of training samples and the *R* ^*2*^ score, respectively. Lines of the same color correspond to the same model, where circular markers denote the training curve and square markers denote the cross-validation curve.

Six algorithms (XGBoost, GBDT, RF, KRR, SVR, and KNN) were compared. Figure 4 shows that training *R* ^*2*^ increased with sample size and then plateaued. Tree-based ensembles (XGBoost, GBDT, and RF) generally outperformed the other methods in both fit and generalization. Under cross validation, most models performed poorly at small sample sizes (N < 80; *R* ^*2*^ < 0.7) with evident overfitting, whereas performance stabilized as sample size increased (N > 120). Across all sample sizes, XGBoost delivered the highest and most stable *R* ^*2*^, with rapid convergence between training and validation curves. GBDT and RF were more variable in the low sample regime and reached lower plateau performance than XGBoost. KNN and KRR showed lower overall accuracy, and SVR exhibited consistently weaker validation performance. Overall, XGBoost provided the most robust and accurate predictions across sample sizes.

#### 3.2.3 Test set and cross-validation performance

As shown in Table 4, across the 50 repeated random 80/20 train–test splits, XGBoost achieved a relatively high mean test-set R^2^ (0.9137 ± 0.0272), together with low MAE (0.0531 ± 0.0075) and RMSE (0.0769 ± 0.0113). The RF model showed performance very close to that of XGBoost, with a test-set R^2^ of 0.9114 ± 0.0266, MAE of 0.0533 ± 0.0077, and RMSE of 0.0781 ± 0.0120. GBDT and SVR also maintained high mean R^2^ values, at 0.9048 ± 0.0330 and 0.9069 ± 0.0256, respectively. In contrast, KNN and KRR showed relatively lower mean R^2^ values and higher error metrics.

**TABLE 4:** Regression metrics of each machine learning algorithm on test sets.

Model	Test		
	R2	MAE	RMSE
XGB	0.9137 ± 0.0272	0.0531 ± 0.0075	0.0769 ± 0.0113
GBDT	0.9048 ± 0.0330	0.0555 ± 0.0090	0.0805 ± 0.0134
RF	0.9114 ± 0.0266	0.0533 ± 0.0077	0.0781 ± 0.0120
KNN	0.8924 ± 0.0273	0.0655 ± 0.0079	0.0863 ± 0.0103
KRR	0.8905 ± 0.0300	0.0653 ± 0.0071	0.0868 ± 0.0105
SVR	0.8630 ± 0.0275	0.0739 ± 0.0083	0.0980 ± 0.0115

In Table 5, XGBoost achieved the highest R^2^ in cross-validation (0.9050, 95% CI: 0.9023–0.9090), together with the lowest MAE (0.0563, 95% CI: 0.0554–0.0571) and RMSE (0.0801, 95% CI: 0.0789–0.0815), indicating the best overall predictive performance. The performance of RF and SVR was relatively close to that of XGBoost, with R^2^ values of 0.9025 for RF and 0.8995 for SVR.

**TABLE 5:** Cross-validation performance (95% CI).

Model	Cross-validation (95%CI)		
	R^2^	MAE	RMSE
XGB	0.905 (0.9023–0.9090)	0.0563 (0.0554–0.0571)	0.0801 (0.0789–0.0815)
GBDT	0.8957 (0.8916–0.8994)	0.0577 (0.0568–0.0587)	0.0839 (0.0826–0.0853)
RF	0.9025 (0.8992–0.9054)	0.0567 (0.0559–0.0576)	0.0820 (0.0810–0.0831)
KNN	0.8847 (0.8806–0.8886)	0.0688 (0.0679–0.0698)	0.0890 (0.0879–0.0902)
KRR	0.8837 (0.8805–0.8867)	0.0666 (0.0658–0.0673)	0.0896 (0.0887–0.0905)
SVR	0.8463 (0.8418–0.8508)	0.0788 (0.0780–0.0796)	0.1038 (0.1027–0.1049)

### 3.3 Collinearity assessment results of input features

As shown in Table 6, the VIF analysis indicated that H/Q had the highest VIF value of 3.462. ITR and KFA showed the next highest VIF values, at 3.311 and 2.948, respectively. The VIF values of the remaining features were all below 2.2. All input biomechanical variables had VIF values below 5, indicating that no serious multicollinearity was present among the selected input features.

**TABLE 6:** Variance inflation factor analysis of input biomechanical variables.

Feature	VIF
H/Q	3.462
ITR	3.311
KFA	2.948
FPA	2.145
HFA	2.101
KVA	1.830
TFA	1.745
ADF	1.699
HAA	1.244

### 3.4 Global SHAP feature importance for the XGBoost model

**FIGURE 5:**
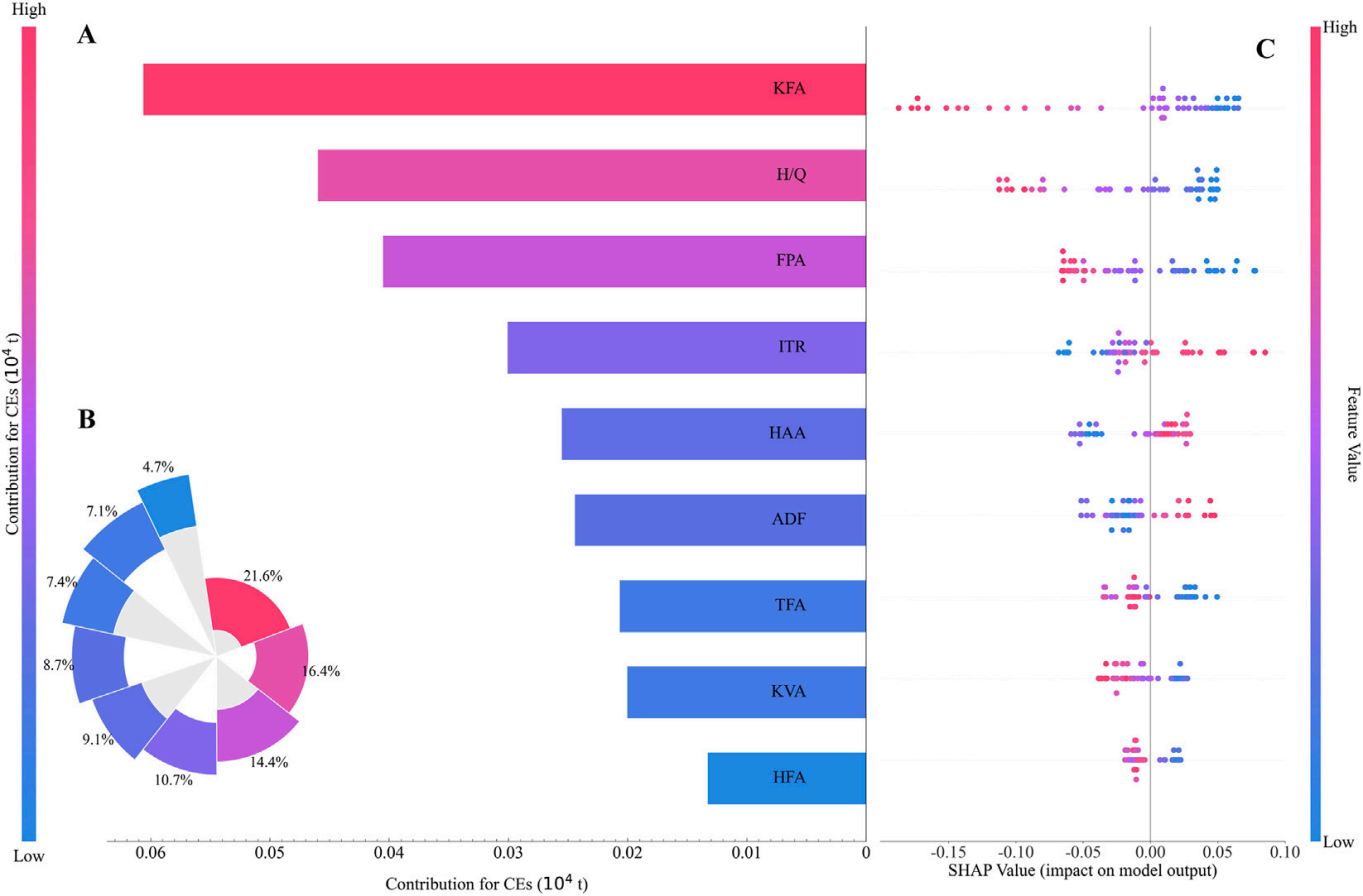
**(A)** Global feature importance ranking based on the contribution of each biomechanical feature to ACL load. **(B)** Percentage contribution of each feature. **(C)** Summary of the effects of features on model outputs. Features on the y-axis are ordered by decreasing importance; the x-axis represents SHAP values, indicating positive or negative contributions to the prediction. Each point corresponds to a sample, with color ranging from blue to red representing low to high feature values.

SHAP values were used to quantify the global contributions of biomechanical features to predicted ACL load. As shown in Figures 5A,B, KFA (21.6%), H/Q (16.4%), and FPA (14.4%) showed the largest contributions and were the primary determinants of ACL loading. ITR (10.7%) and HAA (9.1%) had moderate influence, whereas ADF, TFA, and KVA (each <9%) contributed less. As shown in Figure 5C, the key feature KFA exhibited the widest SHAP spread. Higher values of KFA, H/Q, and FPA were mostly located on the left side with negative SHAP values, suggesting that smaller knee flexion and foot progression angles, together with a lower H/Q ratio, were associated with higher ACL load. In contrast, higher ITR, HAA, and ADF values were mainly distributed on the right side with positive SHAP values, indicating that greater tibial internal rotation, hip adduction, and ankle dorsiflexion were associated with increased ACL load.

**FIGURE 6:**
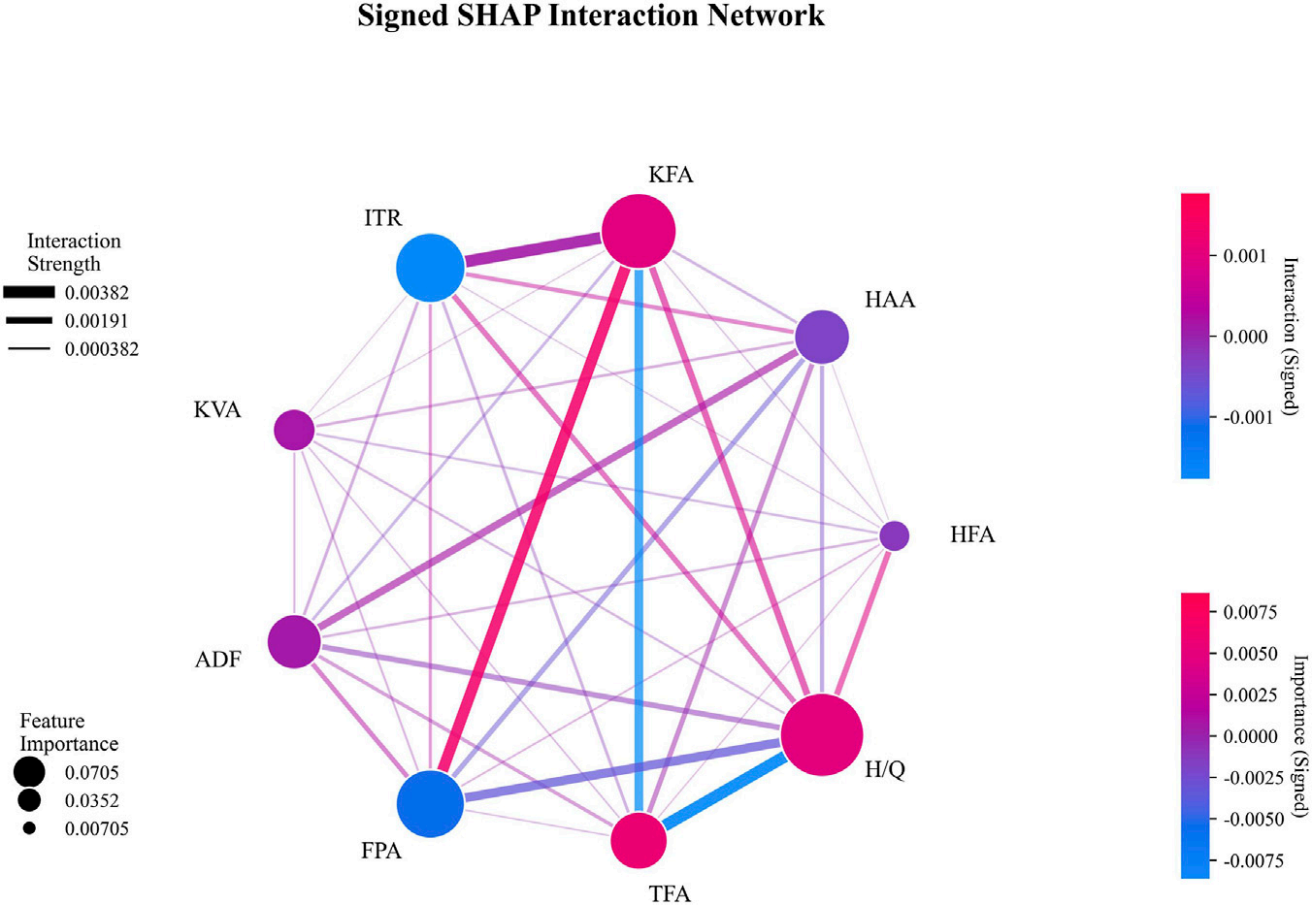
Nodes represent the input features, and node size indicates feature importance; edges represent pairwise SHAP interaction effects between features, with edge width indicating interaction strength and edge color indicating interaction direction. Thicker and redder edges suggest that the corresponding feature combination has a stronger positive joint contribution to the model-predicted ACL loading.

Figure 6 shows that KFA, H/Q, FPA, TFA, and ITR were the main nodes in the interaction network. Among them, KFA–FPA showed the strongest positive interaction effect, while KFA–H/Q, KFA–ITR, and H/Q–HFA also showed certain positive interactions, which tended to increase the model-predicted ACL loading. In contrast, TFA–H/Q and KFA–TFA showed relatively strong negative interactions, which tended to reduce the model-predicted ACL loading.

**FIGURE 7:**
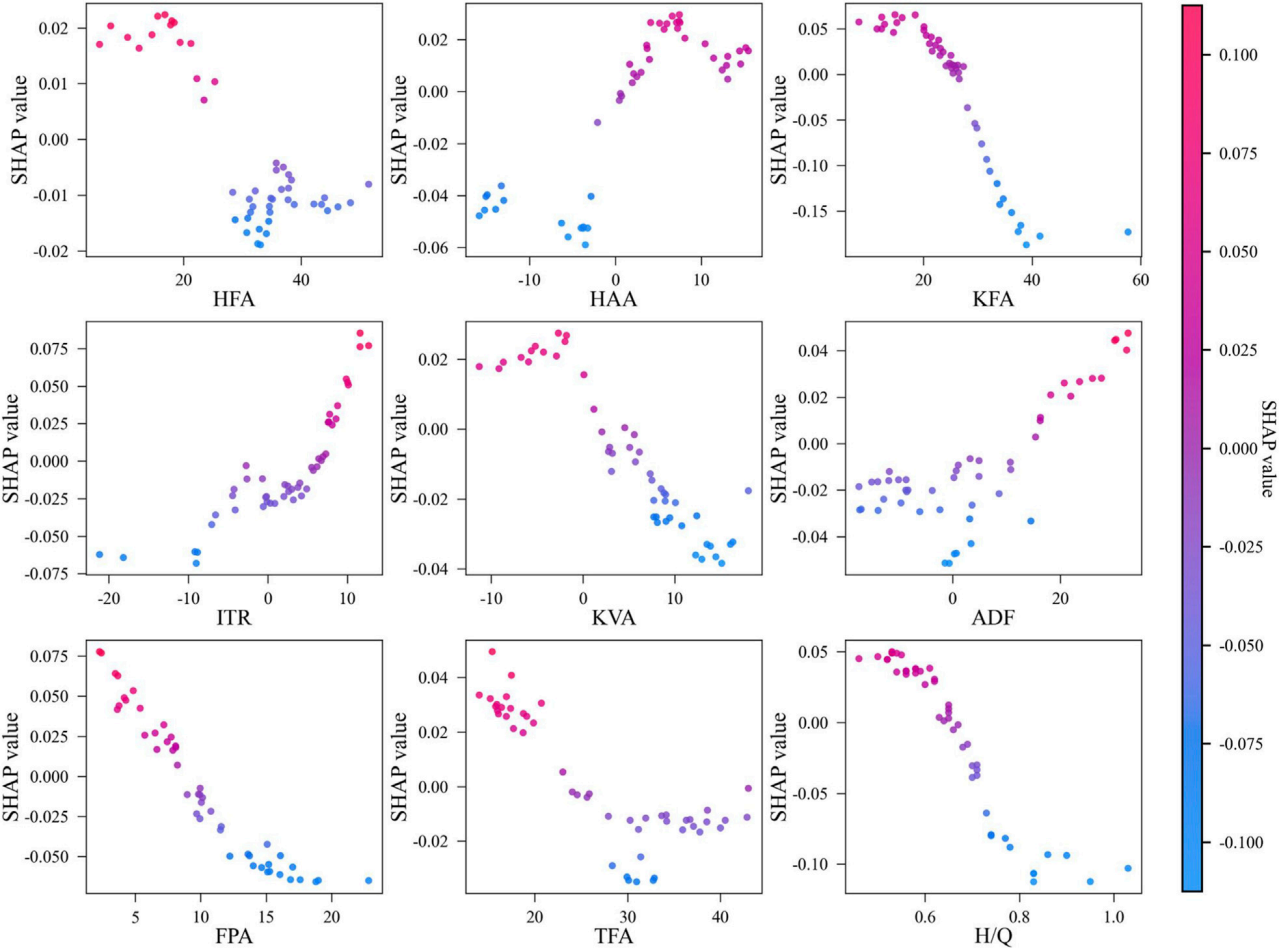
The figure illustrates the interaction between each biomechanical feature and ACL loading. KVA <1.5° was interpreted as a model-identified turning region; beyond this range, a further decrease in KVA indicated a shift toward knee valgus alignment.

Figure 7 shows that KFA, H/Q, FPA, TFA, HFA, and KVA displayed negative relationships with SHAP values. Marked increases in SHAP values were observed when KFA < 27°, TFA < 26°, HFA < 28°, FPA < 9.55°, KVA < 1.5°, and H/Q < 0.66. In contrast, ITR, HAA, and ADF showed positive relationships with SHAP values, with clear increases when ITR > 5.5°, HAA > 0.1°, and ADF > 12°.

### 3.5 Local SHAP explanations for ACL load prediction

**FIGURE 8:**
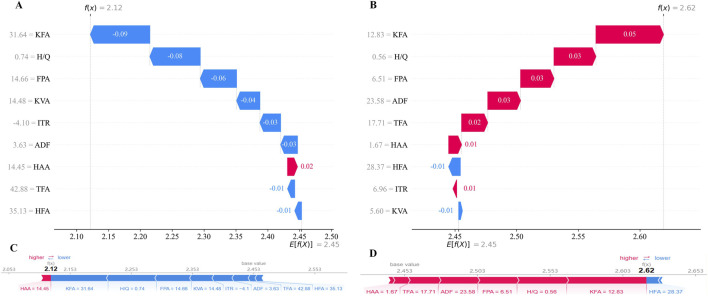
The figure presents the feature importance ranking for high-load and low-load samples alongside the mean predicted ACL load, together with the corresponding feature values for each sample. **(A,B)** show the SHAP waterfall plots for the low-load and high-load samples, respectively, while **(C,D)** show the corresponding force plots with feature values and contribution directions.

Individual predictions were interpreted using SHAP waterfall plots, in which the baseline value, E [f(X)] = 2.45, represents the mean model prediction across all samples. Figures 8A,C show a low-load sample (predicted ACL load = 2.12), in which KFA = 31.64° contributed −0.09, H/Q = 0.74 contributed −0.08, FPA = 14.66° contributed −0.06, KVA = 14.48° contributed −0.04, and both ITR and ADF contributed −0.03, whereas HAA showed a slight positive contribution. Figures 8B, D present a high-load sample (predicted ACL load = 2.62), in which KFA = 12.83° contributed 0.05; H/Q, FPA, and ADF each contributed 0.03; TFA contributed 0.02; and HAA and ITR each contributed 0.01, while HFA and KVA showed slight negative contributions. In the low-load sample, greater KFA, a higher H/Q ratio, and greater FPA were associated with reduced ACL loading. In contrast, in the high-load sample, lower KFA, a lower H/Q ratio, and smaller FPA increased ACL loading.

### 3.6 Web application

**FIGURE 9:**
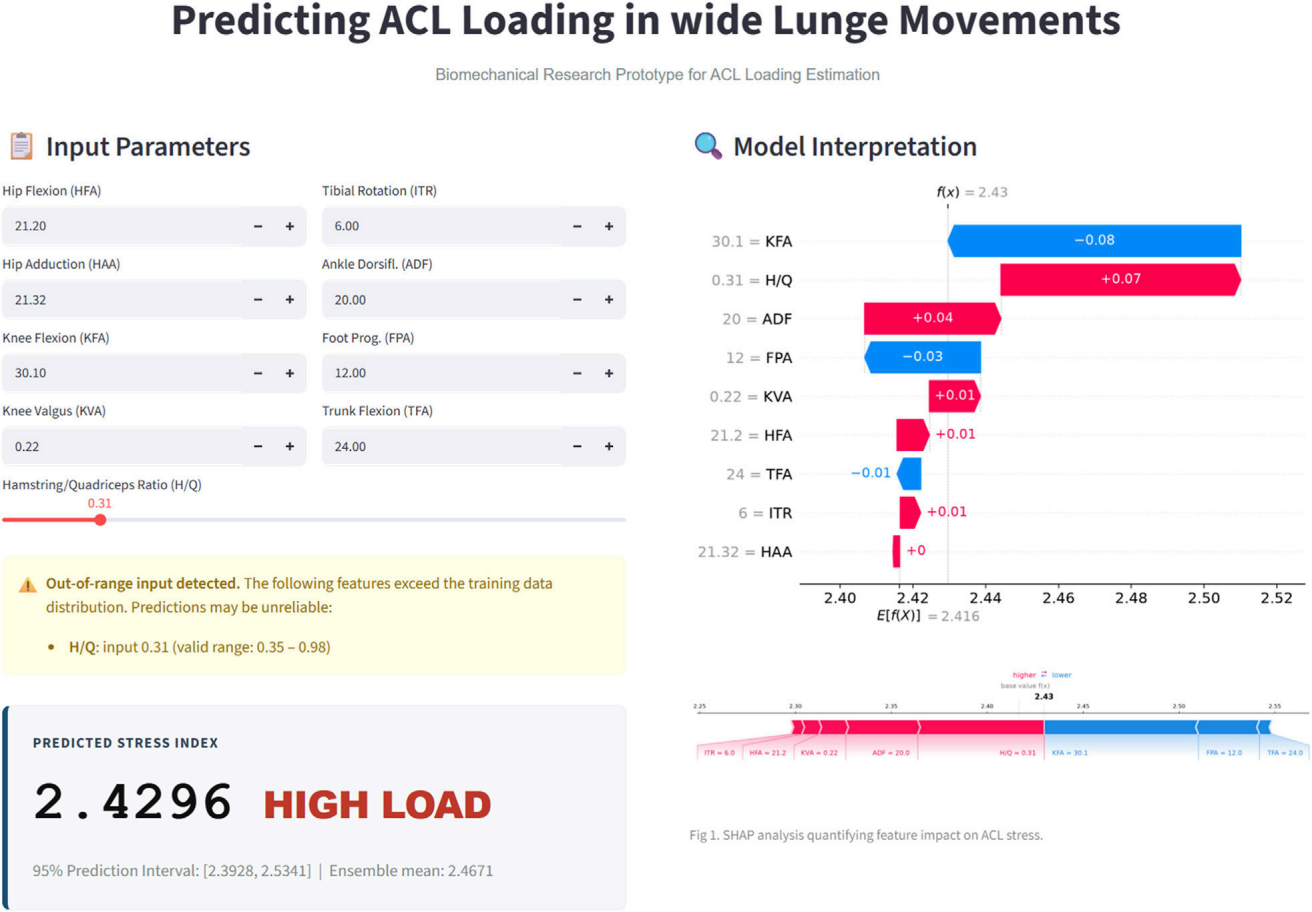
The figure illustrates a web-based interface for ACL load assessment, consisting of two panels. The left panel is used to input nine biomechanical parameters, followed by the output of the predicted ACL load. The right panel provides model interpretability through SHAP-based waterfall and force plots.

Based on the XGBoost regression model, Figure 9 prototype platform for ACL simulated loading prediction and interpretability analysis developed using the Python Streamlit framework. After entering the measured values of the nine features, users can automatically invoke the trained model through the backend. The frontend then synchronously displays the predicted ACL load, individual SHAP force and waterfall plots, allowing a more intuitive visualisation of the contribution of each feature and enhancing the interpretability of the prediction results (https://yu9qvcbw3ffmwtxs8vqtxw.streamlit.app/).

## 4 Discussion

In practical applications, the optimal model should be selected by comparing the data characteristics, computational resources, interpretability, and expected performance metrics of the problem under investigation (Moghadam et al., 2023). The model should also possess good sample efficiency and generalisability, while maintaining robust overall performance when handling high-dimensional non-linear biomechanical features. In this study, a Bayesian optimisation approach based on the Tree-structured Parzen Estimator (TPE) was used for hyperparameter tuning, and the performance of six ML algorithms, including extreme gradient boosting (XGBoost), gradient boosting decision tree (GBDT), random forest (RF), k-nearest neighbours (KNN), kernel ridge regression (KRR), and support vector regression (SVR), was compared. The results showed that XGBoost consistently outperformed the other models in overall performance. During training, the *R* ^*2*^ curve remained stable at a relatively high level, increased gradually, and, after reaching a plateau, the training and cross-validation (CV) curves converged rapidly. These findings suggest that, through its built-in regularisation mechanism and gradient boosting framework, XGBoost can effectively suppress overfitting while maintaining high predictive stability (Chen and Guestrin, 2016). In contrast, GBDT and RF showed weaker learning ability at the small-sample stage, with greater fluctuations in their performance curves. Traditional GBDT lacks the regularisation term incorporated in XGBoost and is therefore more prone to overfitting when sample size is limited. Although RF can reduce variance through its bagging strategy (Zhang et al., 2019), its independently constructed trees make it less capable, under small-sample conditions, of capturing the non-linear relationships among highly coupled features through iterative residual correction, as achieved by XGBoost (Chen and Guestrin, 2016). Therefore, XGBoost demonstrates superior stability when handling small and medium-sized biomechanical datasets and can effectively suppress overfitting.

In model performance evaluation, The three tree-based ensemble methods (XGBoost, GBDT, and RF) showed comparable performance across both five-fold cross-validation and independent test sets, with substantial overlap in their 95% confidence intervals, and all outperformed the remaining models. The relatively higher R^2^ observed on the test set may be attributed to the fact that cross-validation models were trained on only 4/5 of the training data, whereas the final test models were trained on the full training set. These findings suggest that tree-based ensemble models are generally well suited for capturing the nonlinear relationships between kinematic and muscle activation features and the OpenSim-estimated ACL loading during wide lunge tasks. Furthermore, as sample size increased, all models showed gradually converging and stabilising learning curves. Among them, XGBoost, by employing a second-order Taylor expansion of the loss function, was able to capture interactions between features (Chen and Guestrin, 2016), and consistently maintained lower prediction errors and more stable performance across different sample sizes. This indicates strong regularisation capability, effective gradient boosting, and good adaptability to high-dimensional nonlinear biomechanical features. Considering its stable convergence across varying sample sizes, XGBoost was selected as the final model in this study.

In addition, KNN and KRR showed relatively low overall accuracy, whereas SVR exhibited weaker validation-curve performance. As a non-parametric model based on distance metrics, KNN is easily affected by multiple feature dimensions in high-dimensional feature spaces and is generally more advantageous in scenarios with clear data distributions and relatively low dimensionality (Madhavan et al., 2025). In the present study, however, nine feature parameters were included, resulting in a relatively high-dimensional feature space with substantial differences in scale across variables, which made it difficult for KNN to effectively distinguish the similarity of neighbouring samples. KRR and SVR, by contrast, rely on the choice of kernel function when handling non-linear problems. Although kernel functions can theoretically project data into a higher-dimensional space, a fixed kernel structure is difficult to adaptively capture the complex non-linear relationships among feature parameters across different sample sizes in a biomechanical dataset of this complexity (Li et al., 2025; Peng et al., 2020). By comparison, the advantage of XGBoost in handling complex datasets lies in its gradient boosting mechanism and regularisation capacity. As an ensemble tree model, XGBoost demonstrated strong overall adaptability to high-dimensional non-linear biomechanical features and exhibited robust non-linear fitting capability, which supports our hypothesis that XGBoost is superior to the other models for investigating complex multifactorial biomechanical coupling interactions.

ML combined with SHAP provides a game-theoretic framework for interpreting feature importance by attributing each sample-level prediction to individual features, thereby enabling evaluation of global feature importance (Lundberg et al., 2020). In the present study, this approach helped clarify the multifactorial coupling mechanisms of kinematic and electromyographic signals underlying ACL loading. KFA, H/Q, and FPA emerged as the three most influential predictors, followed by ITR and HAA. Among them, KFA was the dominant feature, underscoring the importance of sagittal-plane knee kinematics in ACL loading. This finding is consistent with previous evidence showing that, during jump landing, rapid direction changes, and abrupt stopping, landing with a smaller knee flexion angle substantially increases ACL tension (Englander et al., 2019). Our results further suggest that an insufficient KFA at landing is accompanied by greater quadriceps activation and delayed hamstring activation, thereby increasing anterior shear force on the ACL (Hannah et al., 2014). Reduced KFA may diminish the posterior shear force generated by the hamstrings, thereby limiting their ability to effectively counteract the anterior tibial shear force produced by the quadriceps (Maniar et al., 2022). Mechanistically, quadriceps contraction generates anterior tibial force, whereas eccentric hamstring contraction counteracts this motion, and the dynamic balance between the two determines the net shear force acting at the knee. When H/Q is imbalanced and KFA is simultaneously reduced, anterior tibial translation and anterior shear force increase, which in turn is associated with greater ACL loading (Issaoui et al., 2025). Taken together, the prominence of KFA and H/Q highlights a sagittal-plane knee mechanism captured by the ML model in predicting ACL loading.

The importance of FPA lies in its multiplanar coupling with the hip and knee within the kinetic chain. Previous studies have shown that, through its influence on the ground reaction force, FPA affects the relative position of the knee joint centre and reflects the relationship between distal lower-limb kinetic chain behaviour and proximal joint loading (Nishizawa et al., 2022). Given that wide lunge FPA can alter force-line alignment and redistribute pressure, a certain FPA (<20°) may bring the hip–knee–ankle alignment closer to the sagittal plane and be associated with lower overall knee joint loading (Zhang et al., 2026), thereby forming a coupled pattern in which foot abduction is associated with hip and knee abduction and tibial external rotation. Accordingly, the higher SHAP contribution of FPA may be associated with a proximal–distal coupling mechanism underlying ACL loading, which influences knee joint load distribution. Among the secondary features, the coupling of ITR and HAA suggests a coordinated mechanism between transverse- and frontal-plane motions. Because the ACL is tensioned during tibial internal rotation, excessive ITR is associated with increased the mechanical loading on the ligament fibres (Hodel et al., 2022). An increase in HAA reflects hip abductor control, which may lead to femoral adduction and internal rotation during weight-bearing. Through femur–tibia joint interactions, this may further increase ITR and KVA (Sahabuddin et al., 2021). Forming an adverse coupling pattern consistent with previous biomechanical evidence, characterised by hip adduction, tibial internal rotation, and knee valgus. Although ADF, TFA, KVA, and HFA ranked relatively lower in importance in the present study, they remained integral components of the multifactorial coupling network and, together with the major features identified above, contributed to the regulation of ACL loading. Because ML models can identify multifactorial coupling patterns, they are able to predict ACL values under high-load postures. Therefore, the importance of KFA, H/Q, and FPA interpreted as reflecting the combined effects of kinematic and neuromuscular coupling. By capturing interactions between kinematic and electromyographic signals, ML can identify the mechanisms responsible for increased loading, thereby supporting our hypothesis that increased ACL loading during the wide lunge is driven by multifactorial coupling.

Figure 7 shows non-linear relationships between individual features and ACL loading. KFA, H/Q, FPA, TFA, HFA, and KVA showed negative associations with SHAP values. Specifically, KFA = 27°, TFA = 26°, HFA = 28°, FPA = 9.55°, KVA = 1.5°, and H/Q = 0.66 could be regarded as non-linear turning points at which the model-predicted contribution changed. These findings indicate that when joint angles or the muscle co-activation ratio were below these turning points, their contributions to model-predicted ACL loading tended to increase. Previous studies have suggested that KFA between 0° and 30° and an H/Q ratio below 0.7 during landing are associated with ACL injury (Wu et al., 2010), which is consistent with the turning-point patterns identified by the machine learning model in this study. Earlier studies have also linked ACL injury mechanisms to KFA and H/Q during landing (Englander et al., 2019; Maniar et al., 2022). In the present study, the knee co-activation ratio reflected a critical value in the model explanation for hamstring activation relative to quadriceps activation (H/Q = 0.66). When this ratio was imbalanced, particularly below 0.66, may be consistent with biomechanical explanations such as a weakened protective mechanism against anterior tibial translation. This suggests that the risk associated with the wide lunge may be related to balanced muscle activation. In addition, when FPA was below 9.55°, indicating insufficient foot abduction or a landing pattern closer to foot adduction, the model-predicted loading contribution tended to increase. A moderately increased FPA during landing may alter the force transmission pathway of the lower limb, regulate knee movement patterns, and thereby influence the balance of co-activation between agonist and antagonist muscles around the knee, indicating that a moderate FPA during landing is associated with lower model-estimated ACL loading (Peel et al., 2020). In contrast, ITR, HAA, and ADF showed positive associations with SHAP values. When ITR, HAA, and ADF reached 5.5°, 0.1°, and 12°, respectively, larger tibial internal rotation, hip adduction, and ankle dorsiflexion were suggested to be associated with higher model-predicted ACL loading. In line with previous studies, tibial internal rotation may place the ACL under increased tension, while insufficient hip control and altered ankle buffering strategies may jointly influence lower-limb alignment and load distribution (Lima et al., 2018; Hakukawa et al., 2022). These findings indicate that joint kinematic features do not act independently. Hip adduction may promotes tibial internal rotation, compensatory tibial internal rotation further increases rotational stress on the ACL, and insufficient knee flexion at initial contact may be associated with a high ACL loading state. Together, these results suggest that interactions among multiple features confer substantially greater risk than any single factor alone. Figure 6 further illustrates the synergistic effect of multifactorial coupling on ACL loading. The strongest positive interaction effect between KFA and FPA indicates that the coupling between knee flexion angle and foot progression angle jointly enhanced the model-predicted contribution to increased ACL loading. The positive interaction effects of KFA–H/Q and KFA–ITR suggest that knee flexion, muscle coordination, and tibial rotation formed composite biomechanical features influencing the model prediction of ACL loading. Because limited knee flexion increases anterior tibial shear force, excessive quadriceps activation further amplifies this shear force, and insufficient foot abduction alters force-line alignment, the coupling of these factors with knee flexion may expose the ACL to greater multiplanar combined loading (Beaulieu et al., 2023). The positive interaction effects of KFA–FPA, KFA–H/Q, and KFA–ITR indicate that the coupling between knee flexion angle and foot progression angle, muscle coordination, and tibial rotation formed a high-loading coupling mechanism in the model. This finding indicates that machine learning can identify complex non-linear relationships among biomechanical variables, manifested as coupled features involving knee flexion angle, muscle activation ratio, and foot position.

The SHAP waterfall plot quantifies the specific contribution of each feature to individual ACL load predictions under multi-feature coupling conditions. The baseline value (f(X) = 2.45) represented the mean predicted value across all samples. In the low-load samples shown in Figures 8A,C, the predicted ACL value was f(x) = 2.12. In these samples, the key feature values all lay within the low-load threshold ranges (KFA >27°, H/Q > 0.66, and FPA >9.55°), and thus each feature contributed in the direction of reducing ACL loading. In contrast, the high-load sample shown in Figures 8B,D (f(x) = 2.62) exhibited a typical pattern of abnormal multifactorial coupling (KFA < 27°, H/Q < 0.66, FPA < 9.55°, ADF > 12°, and TFA < 26°), indicating that the coupling of these abnormal factors jointly increased ACL loading (Cortes et al., 2007; Blackburn and Padua, 2008). However, the contributions of ITR and HAA were relatively small in the high-load sample. When these features coexist with sagittal-plane factors across the transverse and frontal planes, their synergistic effects may be far greater than the sum of their individual contributions. Recent experimental studies have reported ACL loading values of approximately 2.3 BW during landing and cutting tasks (Nasseri et al., 2021; Chen et al., 2022), which are close to the values predicted by the XGBoost model in the present study. This supports the credibility of the machine learning-based ACL load predictions. Therefore, machine learning-based prediction of ACL loading during the wide lunge has practical value at the individual level and further highlights the advantage of machine learning in capturing non-linear interactions. To enable rapid assessment of ACL loading, this study further developed an online prediction platform based on the XGBoost-SHAP model. By inputting collected kinematic and surface electromyographic data, users can obtain both load-level classification and SHAP-based visualisation results. This web application can serve as an auxiliary assessment platform under laboratory conditions. Accordingly, with the continued development of portable wearable devices, future systems may incorporate portable EMG sensors and markerless joint-angle measurement systems linked to smartphone-based real-time data acquisition, while feature extraction and other processing are completed in the cloud to enable field-based load prediction (Sédiri et al., 2026; Dergaa et al., 2026). However, before its practical application in training settings, the agreement between portable EMG sensors, markerless systems, and laboratory-based data acquisition should be further validated, and the generalizability and predictive stability of the model in real-world environments should also be evaluated.

It should be noted that ACL loading in this study was calculated by OpenSim based on joint kinematics and ground reaction forces, whereas the muscle co-activation ratio was derived from sEMG data. Therefore, the relatively high R^2^ may partly reflect the model’s recovery of the mapping relationship between kinematics and ligament loading within the musculoskeletal simulation workflow. Nevertheless, the machine learning model can estimate OpenSim-simulated ACL loading using a limited set of biomechanical features. By integrating the SHAP interpretation framework, it further identifies key variables and interaction combinations associated with increased simulated ACL loading, thereby providing a rapid estimation tool and an interpretable interaction-analysis framework for OpenSim-simulated ACL loading.

Despite these findings, several limitations should be acknowledged. First, the feature thresholds and interaction effects identified in this study were derived from a dataset based on a specific movement task. Their generalizability to different sports, sexes, fatigue states, and individual anatomical variations remains to be further validated. Future studies should therefore include female athletes, populations with different competitive levels, and technical movements, and longitudinal cohort designs may be used to examine the clinical validity of these thresholds for injury prediction, thereby further improving the model’s generalizability and scope of application. Second, kinematic data in this study were collected using a conventional laboratory-based optical motion capture system, which provides highly accurate measurements under controlled conditions. Future studies may further adopt markerless motion capture to enable more practical and efficient field-based load assessment and risk prediction.

## 5 Conclusion

By comparing the predictive performance of six ML models for ACL loading, XGBoost was identified as the optimal model. It demonstrated strong regularisation ability, gradient boosting capacity, and good adaptability to high-dimensional non-linear biomechanical features. Machine learning can identify non-linear relationships among complex biomechanical parameters and risk thresholds for key features. The superimposed combination of insufficient knee flexion, quadriceps dominance, and abnormal foot alignment emerged as a high-risk mechanism, indicating that elevated ACL loading results from the synergistic coupling of multiple abnormal factors. Therefore, risk screening should focus on multifactorial coupling effects. For injury-prevention training in the badminton wide lunge, emphasis should be placed on sagittal-plane knee flexion, neuromuscular coordination, particularly optimisation of the H/Q ratio, and control of foot landing posture. These findings further highlight the advantage of machine learning in capturing complex non-linear interactions.

## Data Availability

The original contributions presented in the study are included in the article/Supplementary Material, further inquiries can be directed to the corresponding author.
